# Impact of a Microbial Pest Control Product Containing *Bacillus thuringiensis* on Brood Development and Gut Microbiota of *Apis mellifera* Worker Honey Bees

**DOI:** 10.1007/s00248-022-02004-w

**Published:** 2022-04-07

**Authors:** Charlotte Steinigeweg, Abdulrahim T. Alkassab, Silvio Erler, Hannes Beims, Ina P. Wirtz, Dania Richter, Jens Pistorius

**Affiliations:** 1 Institute of Geoecology, Technische Universität Braunschweig, Braunschweig, Germany; 2 Institute for Bee Protection, Julius Kühn-Institut (JKI) - FederalResearch Centre for Cultivated Plants, Braunschweig, Germany; 3 Institute for Apiculture, Lower Saxony State Office for Consumer Protection and Food Safety (LAVES), Celle, Germany

**Keywords:** Microbial pest control, *Bacillus thuringiensis*, Brood, Gut microbiome, Gut dysbiosis, *Apis mellifera*

## Abstract

To avoid potential adverse side effects of chemical plant protection products, microbial pest control products (MPCP) are commonly applied as biological alternatives. This study aimed to evaluate the biosafety of a MPCP with the active organism *Bacillus thuringiensis* ssp. *aizawai* (strain: ABTS-1857). An in-hive feeding experiment was performed under field-realistic conditions to examine the effect of *B. thuringiensis (B. t.)* on brood development and the bacterial abundance of the core gut microbiome (*Bifidobacterium asteroids*, *Gilliamella apicola*, the group of *Lactobacillus* and *Snodgrasella alvi*) in *Apis mellifera* worker bees. We detected a higher brood termination rate and a non-successful development into worker bees of treated colonies compared to those of the controls. For the gut microbiome, all tested core members showed a significantly lower normalized abundance in bees of the treated colonies than in those of the controls; thus, a general response of the gut microbiome may be assumed. Consequently, colony exposure to *B. t.* strain ABTS-1857 had a negative effect on brood development under field-realistic conditions and caused dysbiosis of the gut microbiome. Further studies with *B. t.*–based products, after field-realistic application in bee attractive crops, are needed to evaluate the potential risk of these MPCPs on honey bees.

## Introduction

While many studies have shown that honey bees are exposed to various environmental and anthropogenic stressors on different biological scales [1], there is recent evidence that the gut microbiome of honey bees is strongly involved in bee health and a rising number of studies focus on the effect of beneficial microbes. The gut of adult honey bees is colonized by a particular microbial community of core phylotypes [2–5] that can be found relatively stable over geographical distances, which is highly affected by the honey bees’ eusocial behaviour, colony organization and division of labour [6, 7]. Although some fundamental properties of the gut microbiome, i.e. the association of beneficial microbes and the ability of their host to buffer against adverse external impacts and to resist pathogens, are revealed, the causes and consequences of gut dysbiosis are not completely decoded [8–11].

Studies on possible side effects of plant protection products (PPPs) examined mainly the effect of chemical products on the survival of bees and the persistence of their gut microbiome. Several experiments with the herbicide glyphosate showed an alteration of intestinal species structure and composition [12–17]. However, when bees were co-exposed to glyphosate and pathogens, controversial results have been observed. Blot et al. [12] found that glyphosate does not significantly enhance the effects of an infection with *Nosema ceranae*, whereas others reported either an increased replication of the pathogen and an impact on the immune response of honey bees or at least a higher mortality of co-exposed individuals, following infection with *N. ceranae*, *Serratia* spp. or the deformed wing virus [13, 15]. Other chemical pesticides affected structure, composition and species richness of the intestinal microbiome and lowered survival of honey bees [18–22]. However, the scale of the effects is depending on the pesticide formulation, test concentrations and species of the tested microbiome. This effect variance becomes critical when considering alternative PPPs, e.g. with microbials as active ingredients. Research on the effect of microbial pest control products (MPCPs) on honey bee health is still developing [23, 24]. In particular, the interaction with the gut microbiome has barely been studied, despite the fact that the use of biological PPPs is increasing and some active ingredients, such as *Bacillus thuringiensis*, exhibit a gut-active mode of action [25, 26].

Based on their rapid degradability under field conditions and their presumed selectivity on insects of the orders Lepidoptera, Coleoptera and Diptera [27–29], MPCP containing the entomopathogenic bacterium *Bacillus thuringiensis* (*B. t.*) are commonly applied in various agricultural systems [25]. The mode of action of *B. t.* is mainly depended on the production of inclusion bodies during the sporulation phase of the bacteria in the insect larva gut, composed of particular insecticidal δ-endotoxins that can be classified into two families Cry- and Cyt-toxins [26–28, 30, 31]. Products with the active organism *Bacillus thuringiensis* ssp. *aizawai* strain ABTS-1857 include several crystal insecticidal proteins, such as Cry1Aa, Cry1Ab, Cry1C and Cry1D and are often used in viticulture and orcharding against caterpillar pests [32]. Applying those in flowering crops, like pome fruit, increases the probability of exposure to non-target organisms including pollinator insects. Furthermore, some reports described a possible production of enterotoxins after germination under very specific culture conditions related to its harboring of enterotoxin genes [33–35], but unlikely under manufacture conditions [32].

As different side effects of *B. t.* on *Drosophila* spp. flies are well known [36–38] and an action of *B. t.* products on other non-target organisms cannot be excluded, frequent exposure of pollinating bees to these products may have unexpected consequences. There are already first results of persistence and distribution of *B. t.* in different hive matrices at colony level either after in-hive feeding [39] or after spray application on oilseed rape [40]. Recent studies have shown differences in behaviour, changes of the midgut physiology and reduced survival of bee adults after treatment with *B. t.*, depending on the *B. t.* strain and exposure route [39, 41–44]. Indirect impacts on bees by potential gut dysbiosis, caused by the consumption of contaminated nectar and pollen, may occur due to the action of *B. t.* in the bee’s gut. Therefore, the aim of our study was (1) to investigate the effect of a MPCP with the active ingredient *B. thuringiensis* subspec. *aizawai* (strain: ABTS-1857) on larval development of *A. mellifera* under field-realistic conditions on colony scale, and (2) to assess the effect of the *B.t.* product on the abundance of selected gut core bacteria species in young worker bees.

## Material and Methods

### Study Design

To investigate the effect of *B. t.* on brood development and abundance of the core gut microbiome (*Bifidobacterium asteroids*, *Gilliamella apicola*, *Lactobacillus* Firm-4/-5 and *Snodgrasella alvi*) in young worker honey bees (*A. mellifera*), an in-hive feeding experiment with a registered MPCP, incl. the active organism *B. thuringiensis* ssp. *aizawai* (strain ABTS-1857), was performed under field-realistic conditions following Oomen et al. [45] and the revision of Lückmann and Schmitzer [46]. The treatment and control groups, each including five colonies with sister queens (about 8,000 workers and a fertile 1-year-old queen), were placed more than 1,000 m apart from each other on agricultural land in the north-east of Braunschweig, Lower Saxony, Germany (52°18′23.6″N, 10°42′08.3″E; 52°18′23.5″N, 10°42′43.3″E). In the experiment, two successive brood cycles were analyzed with a similar design. After caging the queen for 24 h and oviposition, larvae were allowed to develop naturally within their hives. During pupation, combs were removed from the colony and stored in an incubator under dark conditions at 35 ± 2 °C and humidity of 85 ± 5%. Newly emerged bees were color-marked and placed back to their original colonies. Subsequently, marked bees developed their natural intestinal microbiome for 8 days, before being collected for gut preparation and microbiota analysis. In the second brood cycle, colonies were exposed to the MPCP by artificial feeding. Half the maximum field recommended application rate (max. rate of 0.165% product containing 5 × 10^10^ CFU/L in the half rate, depending on the given max. CFU concentrations of 6 × 10^13^ CFU/kg, i.e. 540 g/kg for a comparable product as reported by EFSA (2020) [32]) of the commercial product was mixed in 2 L of 50% *(w/v)* sucrose solution and fed twice to individuals of half of the colonies by using a feeding bag once after oviposition and again after pupation. With this design, both developmental stages (larvae and adults) were exposed to the MPCP and were compared to the control colonies, which were fed only with 50% (*w/v*) sucrose solution.

### Brood Development

The queen of each colony (*n* = 10) was caged on an empty comb for 24 h. Afterwards, each comb contained at least 100 to 300 cells with eggs. This brood comb of each colony was photographed on the following brood fixing days (fixed days corresponding to transition to following developmental stages of worker bee larvae; BFD): 0, 3, 5 and 10, using a PENTAX K-3 camera (ISO 200, diaphragm 8). Photographed brood frames were analyzed using the program HiveAnalyzer (Visionalytics, Höferlin, Benjamin & Höferlin, Markus GbR, Pleidelsheim, Germany). In brief, on the image of the first BFD, 100 to 300 brood cells were marked and their development on the following BFDs was assessed via a brood index. For every larval instar, a brood index was defined and the brood termination rate and pupation rate were determined, according to [47].

### Gut Preparation and Microbiota Analysis

Whole guts were removed from 40 individuals of each group and colony and placed, in pools of five guts, in sterile lysis tubes (innuSPEED Lysis Tube P, Analytik Jena) with ceramic beads (2.4–2.8 mm) for sample homogenization in 200 µL 154 mM NaCl. Finally, eight pools of each colony were available for further extraction and stored at − 80 °C until DNA analysis.

Genomic DNA was isolated by using the NucleoMag® VET Kit (Macherey–Nagel) on an epMotion®5075 system (Eppendorf). DNA was finally eluted in 50 µL elution buffer. Multiplexed qPCRs, microbial targets and *Apis*-reference gene target, each was performed in an AriaMX Real-Time PCR System (Agilent Technologies). Reaction volumes (10 µL) included 1.0 µL template, 5.0 µL LUNA® Universal Probe qPCR Master Mix (New England Biolabs), 0.4 µL primer (10 mM), 0.2 µL probe (10 µM) (Table 1) and 3.0 µL nuclease-free water. Reactions were run after an initial denaturation at 95 °C for 60 s in 45 cycles of denaturation at 95 °C for 15 s, annealing and extension at 60 °C for 30 s, including a plate read at the end of each extension step. Probes for each target were designed on the targets’ in silico sequences by Primer3web (version 4.1.0) [48].

**Table 1:** Overview on primers and probes for gut microbiome quantification using qPCR

Target organism	Oligonucleotide	Sequence (5′-3′)	Reference
*A. mellifera*	prPE172	TGC CAA CAC TGT CCT TTC TG	Engel et al*.* 2015
	prPE173	AGA ATT GAC CCA CCA ATC CA	
	pApis^1^	ACT GCC CTA GCA CCA TCC ACC ATG A	This study
*B. asteroides*	Bifi-F2	TCG CGT CYG GTG TGA AAG	Rouze et al*.* 2019
	Bifi-R2	CCA CAT CCA GCR TCC AC	
	pBifi^1^	CGG TGT AAC GGT GGA ATG TG	This study
*G. apicola*	Gil-nest-fw	CGT TAA TTA CAG AAG AAG CAC CG	This study
	Gil-nest-rv	AGT GCA ATT CCT AGG TTG AGC	
	pGill^1^	AGG GTG CGA GCG TTA ATC GGA ATG	This study
*Lactobacillus* Firm-4/-5	Lact_new_fw	AGC AAC GCG AAG AAC CTT AC	This study
	Lact_new_rv	AAT AAG GGT TGC GCT CGT TG	
	pLact^1^	GCT CGT GTC GTG AGA TGT TG	This study
*S. alvi*	Beta-1009-qtF	CTT AGA GAT AGG AGA GTG	Rouze et al*.* 2019
	Beta-1115-qtR	AAT GAT GGC AAC TAA TGA CAA	
	pSnod^1^	ATG GCT GTC GTC AGC TCG TG	This study

^1^Oligonucleotides were dual labeled to act as qPCR probe

qPCRs were conducted in technical triplicates per sample and target. *C*_q_ values were filtered for values between 11 and 35. All others were seen as outliers and removed from the data set. The relative bacterial abundance was determined for each bee pool sample using the *C*_q_ of each bacterium and the corresponding *C*_q_ of the *A. mellifera* reference gene (β-actin), averaged among technical replicates [49]*.* To correct for time (brood cycle) and colony variance, data of single bee’s relative bacterial abundance of each colony, estimated from the second brood cycle (control vs. treatment), were normalized to the relative bacterial abundance of the corresponding colony (using the geometric mean among the eight pools), measured from the first brood cycle (without any treatment).

Presence and quantity of *B. t*. were measured using the bee gut homogenates, serial dilution plate counting of bacterial colony-forming units (CFUs) and *B. t*.–specific PCR following the protocols in [37, 40].

### Statistical Analysis

For brood development, larval transitions of exposed and control bees were compared with a *t*-test for each BFD. The level of significance was set to 0.05 for all tests and the statistical analyses were performed using R (R Core Team 2019, version 3.6.1).

Normalized qPCR data did not fulfil criteria for data normality (Shapiro–Wilk’s test) and homoscedasticity (Levene’s test). To test for changes of the normalized bacterial abundances of *B. asteroides*, *G. apicola*, *Lactobacillus* Firm-4/-5 and *S. alvi* in the gut of young worker bees between treatment groups, pair-wise Mann–Whitney *U* tests with post hoc Bonferroni-Holm correction were used.

## Results

### Effect of Bacillus thuringiensis on Brood Development

**Fig. 1:**
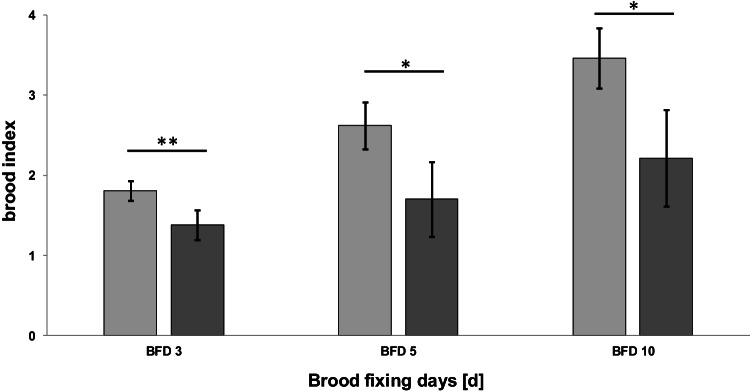
Brood development of honey bee larvae exposed to *B. thuringiensis* product, strain ABTS-1857, (in dark grey) compared to the control (in light grey) (*N* = 5 colonies per treatment; *n* = 100–300 brood cells per colony; *t*-test; *p < 0.05, **p < 0.01). The indices reflect the expected developmental stage at each date, where brood index of 1 for eggs, 2 for young larvae, 3 for old larvae, 4 for pupae is used

**Fig. 2:**
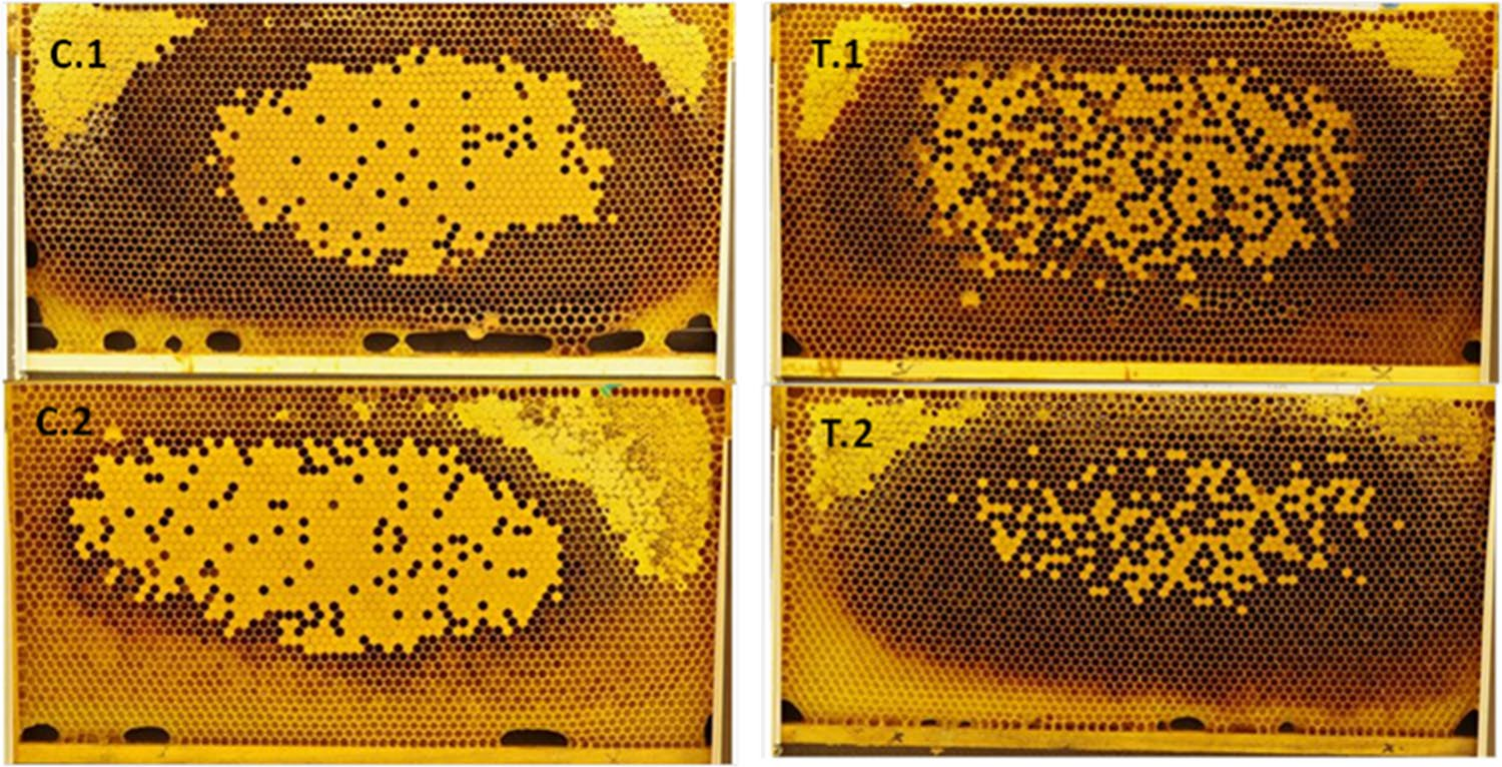
Brood development on the same day in treated compared to untreated colonies. Inconsistent development of brood cells with many gaps is shown in the treated colonies. C.1 and C.2 are control colonies; T.1 and T.2 are treated colonies

To evaluate the effect of the *B. t.* product on the development of larvae under field-realistic conditions, an in-hive feeding experiment was conducted. Until pupation, the brood indices of every larval stage were significantly lower in colonies exposed to *B. thuringiensis* than in untreated colonies (*N* = 5 colonies per treatment; *n* = 100–300 brood cells per colony; BFD 3: *t* = 3.85, *p* = 0.006; BFD 5: *t* = 3.33, *p* = 0.013; BFD 10: *t* = 3.52, *p* = 0.010; Fig. 1). On average, the termination rate (the number of non-successfully developed brood) at BFD 10 was 44.65 ± 16.80% in the treated colonies and 13.40 ± 10.61% in the untreated colonies. Thus, the development of the brood in treated colonies was inhibited and the number of emerged worker bees was expected to be reduced by slightly less than half in colonies exposed to *B. t.* Furthermore, we observed that the brood nest of the treated colonies was remarkably full of gaps and was inconsistent (Fig. 2).

### Effect of Bacillus thuringiensis on the Abundance of Core Gut Bacteria

**Fig. 3:**
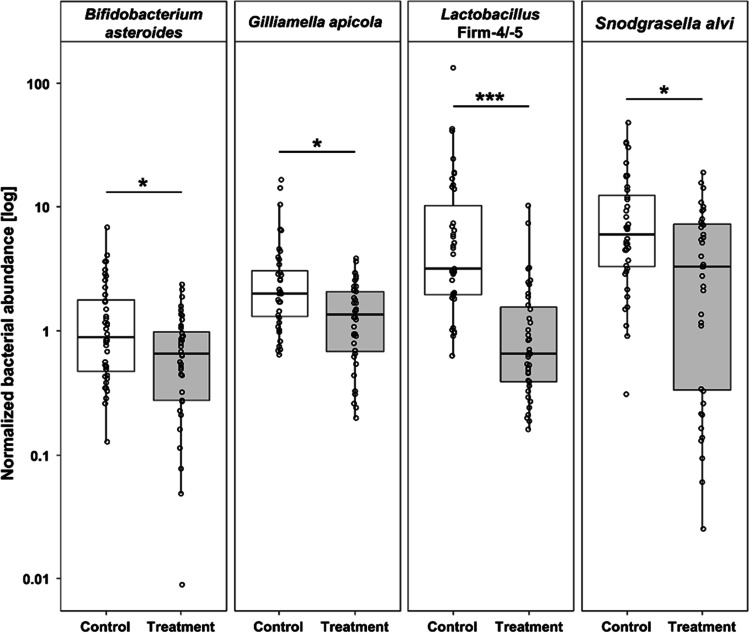
Normalized bacterial abundance (log-scale) for *Bifidobacterium asteroides*, *Gilliamella apicola*, *Lactobacillus* Firm-4/-5 and *Snodgrasella alvi* determined in colonies treated with a commercial *B. thuringiensis* product (Treatment, grey boxes) and control colonies receiving control food (Control, white boxes). Box-plots with boxes for the inter-quartile range, whisker for the 1.5 interquartile range and empty circles showing single raw data points. The values measured after the second brood cycle were normalized to their corresponding colony values of the first brood cycle. A value of 1 indicates no change in bacterial abundance between the two brood cycles. Asterisks indicate significant differences (*p < 0.05, ***p < 0.001) between treatment groups

To determine the impact of the *B. t.* product on the abundance of selected gut microbiome bacteria under field-realistic conditions, young adult honey bees of the in-hive feeding experiment were collected and their guts analyzed via qPCR. In all cases, the normalized bacterial abundance was strongly reduced in the treated colonies (*B. asteroides*: *U* = 566, *n* = 80, *p* = 0.025; *G. apicola*: *U* = 496, *n* = 80, *p* = 0.011; *Lactobacillus* Firm-4/-5: *U* = 190, *n* = 78, *p* < 0.001; *S. alvi*: *U* = 489, *n* = 78, *p* = 0.014; Fig. 3). The exposure of honey bee colonies to the commercial *B. t.* product had a significant adverse impact on the core microbiome of nurse worker bees.

Estimating the quantities of *B. t.* CFUs in the honey bee colonies during the first brood cycle (i.e. before the exposure phase) revealed complete absence of this bacterial species, which indicated that all experimental colonies were not exposed naturally to *B. t.* For the second brood cycle (i.e. during the exposure phase), colonies treated with the MPCP had 6855.30 times higher CFUs/ml bee homogenate (treatment: median 3.59 × 10^6^ CFUs/ml, control: median 5.24 × 10^2^ CFUs/ml) than the untreated controls.

## Discussion

Based on their foraging activity, worker honey bees are potentially vulnerable to be exposed to MPCPs, including *B. t.*. After in-field application, honey bee workers may transfer bacteria to and distribute them within their hives, where the fate of *B. t.* is reported by Alkassab et al. [40]. In general, produced *δ-*endotoxins are known to be rapidly degradable and endospores are inactivated when applied as a spray and exposed to UV radiation [32]. However, spore loads decreased over time in nectar (honey stomach), pollen pellets and adult bees under field conditions, whereas loads increased under colony conditions in larvae or stayed unchanged in stored matrices (stored nectar, bee bread) [39, 40]. Constant temperature, humidity and absent radiation can be crucial factors for *B. t.* persistence and development in hives. To investigate possible effects of a product containing the *B.t.* strain ABTS-1857 on larvae during a brood cycle in the bee hive, we performed an in-hive feeding experiment and recorded the bees’ development photographically. The results indicate a significantly increased brood termination rate in the treated colonies compared to untreated colonies. This effect was mostly observed during the larval stage, where the larvae exposed to *B. t.* seemed to have a lower probability to successfully complete their development and reach pupation. This agrees with our observations from previous in vitro laboratory experiments, where those larvae that reached the pupation phase after chronic exposure to *B. t.* strain ABTS-1857, were able to develop into imagines [39]. Metamorphosis might explain this observation, as pupating bees lose their erratic microbiome when shedding their gut lining [2].

Concerning the toxic action in the insect gut, *B. t.* may affect the gut microbiome of adult honey bees. However, a healthy gut microbiome is assumed to be essential for bee health [10, 11, 15, 50, 51]. The development of a stable gut microbiome depends on different environmental, eusocial conditions, and is associated with the occurrence of a core gut community in honey bees [3–7]. Kwong and Moran [2] reviewed the findings of gut communities in social bees, emphasizing the importance of the transmission routes for the establishment of a stable gut microbiome. Newly emerged honey bee adults show germ-free intestinal systems [2, 52, 53]. Kwong and Moran [2] assumed that young adults acquire an initial inoculation of residual gut symbionts from the frame surface, when chewing out of their cell on their own. At this time, an infection with *B. t.* distributed in the hive cannot be avoided and may disturb the subsequent establishment of the normal gut community. Generally, the microbial community establishes within the first 8 days after emergence, beginning relatively erratic and small, via oral and faecal transmission through nest mates and hive material [2, 53, 54]. Thus, the transmission of *B. t.*, not only by contact with nesting material, but also by exchange between individuals and behavioural stages may be important for an exposure. Additionally, the tested product is gut active. An interaction with the gut microbiome of honey bees is therefore highly probable but has not been proven yet. Such possible interactions were previously shown between various chemical PPPs and the gut microbiome of young worker bees. Characteristics, such as the abundance, composition or species richness of the bacterial gut microbiome of honey bees, were altered after the exposure to the herbicide glyphosate [e.g. 12–17. Besides, honey bees exposed to chemical PPPs showed a higher susceptibility, when co-infected with *N. ceranae* or pathogens of the genus *Serratia* [12, 13, 15, 17]. Similar susceptibilities were described by Rayman et al. [51] after the treatment with an antibiotic and a subsequent infection with a *Serratia*-pathogen. The colonization rate of *Serratia* in bee guts with a weakened gut microbiome was significantly increased. However, the effects of chemical pesticides and MPCPs can be very different, making extrapolation between the two types uncertain. Therefore, analogies of *B. t.* (strain ABTS-1857) and its spores can only be expected. In order to obtain further data on a possible altered composition of core bacterial species in honey bee guts, we investigated selected species of the gut microbiome of young honey bee workers after two exposures with *B. t.* All tested bacterial species of the core microbiome were present in bees from treated as well as in those from control colonies. The relative abundance of the tested bacteria was significantly reduced in bees exposed to *B. t.* compared to the control. This indicates a gut microbiome dysbiosis phenomenon and can be cautiously compared to observations in bees after exposure to chemical PPPs composition [18]. The dysbiosis might be the result of the bacteria replication within the bee’s digestive tract. Measuring *B. t.* CFUs/ml from the honey bees gut homogenates showed a strong presence of *B.t.* in the gut system and may support a trade-off between core symbiotic bacteria and the active organism of the MPCP, both fighting for nutritional resources and habitat space. The potential effects of the gut microbiome dysbiosis on the bee’s physiology and survival remain speculative at the current stage.

Besides a general effect of *B. t.* on the bees’ gut microbiome, a gut bacteria species-specific response to a contamination with a PPP or possibly a MPCP may be presumed. Depending on the higher susceptibility of *S. alvi* to glyphosate because of encoded EPSPS genes, Motta et al. [15] inferred alternative mechanisms of glyphosate resistance typical for this species, so a species-specific reaction to different potential hazards in the gut microbiome is likely. This is distantly supported by the susceptibility of the bacterial, but not of the fungal microbiome in bees to an exposure to coumaphos, tau-fluvalinate and chlorothalonil [55]. Finally, Liu et al. [18] found that the gut of middle-aged bees (i.e. on day 13) recovered from dysbiosis, caused by a thiacloprid exposure. Those results suggest that a reconditioning of the gut microbiome may be possible. Investigations on reconditioning should be carried out in future with chemical as well as with biological PPPs in order to generate a sufficient and comparable data basis.

The effect of external factors, such as MPCPs, on the microbiome may be subject to particular temporal or spatial conditions, such as the bee’s age, product formulation, exposure route [42, 44] or local environment. Despite our observations with strain ABTS-1857, a laboratory study with the toxin *B. t.* Cry1Ie of transgenic Cry1Ie maize found no differences on the diversity of midgut bacteria in bees [56]. Probably, the mode of action is different in case of the *B. t.* product containing the bacterial spores of the strain ABTS-1857 and their Cry-toxins. The controversial discussion about *B. t.* and bees in the literature points to a dependence on formulation and *B. t.* strains, and highlights significant data gaps that require further studies [24]. Thus, our results (based on strain ABTS-1857) are not sufficient evidence to prove a general negative impact of *B. t.* on the honey bees’ gut microbiome.

## Conclusion

Our results showed a clear adverse impact of *B. thuringiensis* subspec. *aizawai* (strain: ABTS-1857) on the larval development after in-hive feeding. Furthermore, a dysbiosis of the gut bacteria *B. asteroides*, *G. apicola*, *Lactobacillus* Firm-4 and -5 and *S. alvi* in young worker bees was demonstrated. However, the variety of factors probably driving the action of *B. t.* products and the response of the individual gut microbiome makes a final evaluation tough. In further investigations, potential competitive interactions between *B. t.* and the gut microbiome might be analyzed. Moreover, different bacterial as well as fungal species of the honey bees gut microbiome should be considered.

## Data Availability

The datasets used and/or analyzed during the current study are available from the corresponding author on reasonable request.
